# A Minireview Exploring the Interplay of the Muscle-Gut-Brain (MGB) Axis to Improve Knowledge on Mental Disorders: Implications for Clinical Neuroscience Research and Therapeutics

**DOI:** 10.1155/2022/8806009

**Published:** 2022-09-15

**Authors:** Davide Maria Cammisuli, Jonathan Fusi, Giorgia Scarfò, Simona Daniele, Gianluca Castelnuovo, Ferdinando Franzoni

**Affiliations:** ^1^Department of Psychology, Catholic University, Milan, Italy; ^2^Department of Clinical and Experimental Medicine, University of Pisa, Italy; ^3^Department of Pharmacy, University of Pisa, Italy; ^4^Psychology Research Laboratory, Istituto Auxologico Italiano, IRCCS, Milan and Piancavallo (VB), Italy

## Abstract

What benefit might emerge from connecting clinical neuroscience with microbiology and exercise science? What about the influence of the muscle-gut-brain (MGB) axis on mental health? The gut microbiota colonizes the intestinal tract and plays a pivotal role in digestion, production of vitamins and immune system development, but it is also able to exert a particular effect on psychological well-being and appears to play a critical role in regulating several muscle metabolic pathways. Endogenous and exogenous factors may cause dysbiosis, with relevant consequences on the composition and function of the gut microbiota that may also modulate muscle responses to exercise. The capacity of specific psychobiotics in ameliorating mental health as complementary strategies has been recently suggested as a novel treatment for some neuropsychiatric diseases. Moreover, physical exercise can modify qualitative and quantitative composition of the gut microbiota and alleviate certain psychopathological symptoms. In this minireview, we documented evidence about the impact of the MGB axis on mental health, which currently appears to be a possible target in the context of a multidimensional intervention mainly including pharmacological and psychotherapeutic treatments, especially for depressive mood.

## 1. Introduction

From a historical point of view, the pivotal role of the gut microbiota on an individual's health was first conceived by the Russian biologist E. Metchnikoff, who described some health benefits in a population of poor Bulgarians connected to the consumption of lactic acid bacteria in fermented milk [1].

On one side, *microbiota* refers to a specific population of organisms (i.e., bacteria, yeasts, and parasites) colonizing the skin, the respiratory, the uro-genital, and the gastrointestinal tract, where the majority of the population lives. The human gut is a complex, dynamic, and heterogeneous system which exert a marked influence on the host during homeostasis and disease. It contains 10^13^-10^14^ microorganisms, and its weight is about one kilogram in the adult, with the majority of bacteria residing in the colon [2]. Through physiological functions, the microbiota can offer specific benefits to the host, such as strengthening gut integrity or shaping the intestinal epithelium, harvesting energy, protecting against pathogens, and regulating immunity [3]. In healthy adults, two bacterial phyla, *Bacteroidetes* and *Firmicutes*, dominate the gut bacterial composition, with smaller amounts of *Actinobacteria*, *Proteobacteria*, and *Verrucomicrobia* [2]. Alterations that affect the commensal flora impair microbial homeostasis and generate a condition called “*dysbiosis*”; particularly, gut *dysbiosis* is characterized by a significant decrease of *Bacteroidetes* and *Lactobacilli* [4]. In a similar way, *Lactobacillus* abundance is predominant in other body districts, including vagina and endometrium [5], and even in the latter, eubiosis exists if the percentage of endometrial *Lactobacilli* is greater than 90% [6].

On the other side, the *microbiome* consists of the genes that microbial cells harbor [7]. It comprises all the genetic material within a microbiota, the whole collection of microorganisms in a definite *situs*, in such a case, the human gut. This has been defined by some researchers as the “*metagenome of the microbiota*”, too [8].

Evidence from literature documented that the alteration of the native microbial intestinal florae is being invoked in nutrition, human metabolism, direct host defense, immunological development, physiological and pathological aging, and even psychiatric disorders [9]. Starting from this assumption, microbiota manipulation may represent a promising tool as adjunct therapy for treating specific mental illnesses and their associated symptoms [10].

Moreover, the impact of the gut microbiota on skeletal muscle function and quality in terms of energy, neuromuscular connectivity, mitochondrial function, and endocrine and insulin resistance, has recently been the focus of some research attempts [11]. The gut microbiota may represent a challenging new therapeutic opportunity and advances in the field of exercise science may enrich the heritage of clinical neuroscience applied to psychiatric disorders. Studies reporting experiments on the gut microbiota intervention documented that specific probiotics have the potential to interact with the brain and exert a positive bacteria-mental functioning relationship [12]. Altered gut microbial profiles have been described in several psychiatric disorders and psychobiotics are currently employed as adjunct treatment to pharmacological and psychotherapeutic interventions. Many of these effects appear to be specific, suggesting a potential role of certain probiotic strains. Further, physical exercise inducing microbial changes with release of neuroendocrine factors may lower inflammatory and oxidative stress of the brain [13].

This mini-review briefly summarizes the progress of research on the muscle-gut-brain (MGB) axis highlighting the role of psychobiotics and physical activity in modulating the response of the microbiota and its effects on mental health, and discusses implications for clinical neuroscience research and therapeutics.

## 2. The MGB Axis: Communication Links and Role of Physical Activity in the Mutual Relationship between the Gut and the Skeletal Muscles

**Figure 1:**
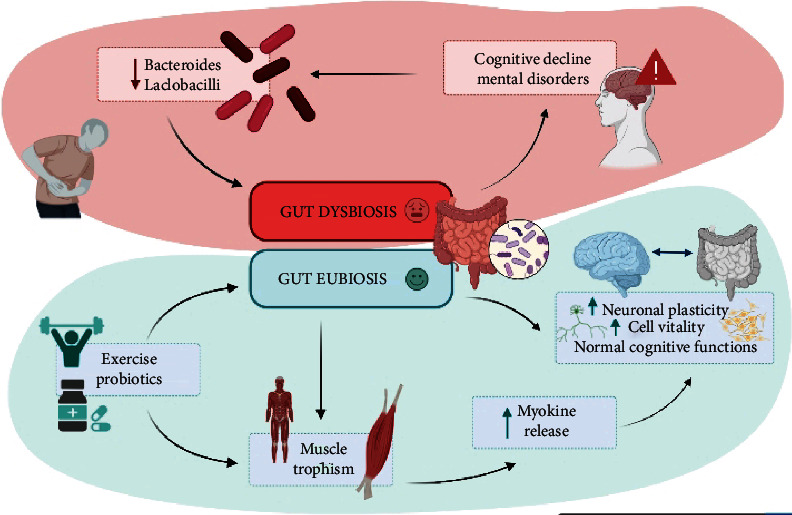
A representation of the gut eubiosis/dysbiosis effects on brain and muscle activities.

As well as regulating brain functions, the gut microbiota affects the skeletal muscle functioning. The graphical representation (Figure 1) depicts gut eubiosis and dysbiosis. In particular, intestinal *eubiosis*, conceived as the balance of the intestinal microbial ecosystem, favors the integrity of the gut barrier and prevents the translocation of liposaccharides (LPS) and other harmful products in the bloodstream, with positive effects on systemic inflammation which could alter muscle metabolism [14–16]. On the other hand, intestinal *dysbiosis*, an ecosystem where “*good*” and “*bad*” bacteria do not live in mutual harmony, [1] is also responsible for a decreased activation of AMPK (i.e., AMP-activated protein kinase) and PGC-1*α* (i.e., proliferator-activated receptor coactivator-1) signaling pathways, which are at the basis of autophagy mechanisms. Autophagy, in fact, is fundamental for the skeletal muscles to remove older organelles and myocytes and to preserve muscle functions [17]. Moreover, an impaired autophagy stimulates inflammation and oxidative stress that negatively affects muscle vitality [18].

An altered gut microbiota also affects insulin-like growth factor-1 (i.e., IGF-1) release. IGF-1 usually activates phosphatidylinositol 3-kinase (i.e., PI3K-AKT) signaling pathway that inhibits mRNA transcription and muscle protein synthesis [19]. In murine models, the lack of a gut microbiota decreases levels of IGF-1 reducing the transcription of genes fundamental for efficient mitochondrial functions within the skeletal muscles [20]. Therefore, intestinal dysbiosis promotes inflammation, oxidative stress, and alters muscle anabolism and mitochondria impairing muscle vitality [11].

In recent years, the interaction between the gut microbiota and the muscles has been receiving considerable attention from the scientific community [21]. It is now well established that the integrity of the muscular system correlates with regular physical activity. On the basis of such evidence, an attempt has been made to establish how the intestinal microbiota may influence the muscular system, or whether physical activity may lead to intestinal eubiosis or dysbiosis.

The positive interaction between physical activity and the gut microbiota is highlighted by the studies of Santacroce et al. [22] and Manders et al. [23], in which it is observed that a low amount of physical activity can induce a reduction in the risk of colon cancer, diverticulosis, and irritable bowel syndrome (IBS). These results are confirmed in the study of Monda et al. [24] documenting how regular physical activity reduces inflammation in the intestine. In their studies, Petersen et al. [25] and Scheiman et al. [26] showed that athletes have a greater biodiversity of the fecal microbiota and also a presence of mycobacterium correlated with the health status. Physical exercise modulates not only the expression of the gut microbiota in terms of microorganisms, but also the production of immunoglobulin A (i.e., IgA) and the reduction of B-cells and T-CD4 in murine models. Such modifications suggest that the gut microbiota also has immunomodulatory functions [27]. However, prolonged and strenuous exercise increases intestinal permeability. Such a mechanism causes a passage of the bacteria from the colon with the consequent risk of gastrointestinal problems [28]. When analyzing the scientific literature, it is always difficult to understand which type of physical activity (e.g., endurance exercise, resistance training exercise, acute or chronic exercise sessions, etc.) induces better changes [29]. Endurance exercise, that is a kind of cardiovascular exercise performed over a prolonged period of time [30], induces a number of major adaptations such as capillary neogenesis, mitochondrial biogenesis, and increased cardiofitness. In addition, endurance training increases *Lactobacillus*, *Bifidobacterium*, and *Blautia coccoides-Eubacterium rectale* species, while a decrease of *Clostridium* and *Enterococcus* has been found in a rat model [31].

Clarke et al. [32] showed that athletes (i.e., rugby players) had greater variability of the gut microbiota than sedentary individuals. The greater variability is the basis for an improved overall health. *Firmicutes* and *Lactobacillales* are two classes of microbes that seem to be affected by positive changes induced by endurance exercise (i.e., ability to last) [33]. Few studies pointing out the relationship between resistance training (i.e., all exercises in which a force is required to overcome a resistance) and the composition of the gut microbiota are present in literature [34]. In a recent study by Castro et al. [35], it was observed that 12 weeks of resistance training promoted the diversity and the composition of the gut microbiota in rats. In the trained group, an abundance of *Pseudomonas* and, in contrast, a decrease in *Serratia* and *Comamonas* were observed. Subsequently, in a study conducted in a human model by Moore et al. [36], it was observed that 6 weeks of resistance training can improve the integrity of the intestinal barrier in a group of elderly subjects by modulating the population of intestinal microbes. In conclusion, it should be noted that the relationship between physical activity and microbiota is inverse. In fact, some studies have shown that a correct composition of the intestinal microbiota (or *eubiosis*) improves athletic performance [37–39]. Indeed, it was observed that sport performance (i.e. endurance swimming) was better in specific pathogens (SPF) and *Bacteroides fragilis* mice than in germ-free mice. This result suggests that the composition of the gut microbiota may be crucial for athletic performance. Moreover, the study also showed a possible improvement of antioxidant systems in SPF mice, linked to an increased plasmatic levels of glutathione peroxidase and catalase [40]. In this regard, it has to be considered that intestinal microbiota exerts beneficial effects on the oxidative stress status; several microorganisms have antioxidant properties since they are able to improve the expression of antioxidant enzymes as well as controlling the release of proinflammatory cytokines [41]. The abundance of *Lactobacillu*s species enhances the activity of superoxide dismutase (i.e., SOD), the levels of glutathione and the scavenging activity against hydroxyl radicals [42]. In contrast, *Escherichia coli* and *Enterococcus* abundance make organisms susceptible to oxidative stress damages [43]. Considering the above, in addition to a proper balanced diet, a moderate and regular exercise can modulate microbial species within the gastrointestinal tract, that, in turn, regulate inflammation and oxidative stress, with positive implications both on muscle performance [44] and brain health [43]. Indeed, muscle trophism is fundamental to ensure, in response to exercise, the release of hormone-like molecules called myokines, such as cathepsin B, FNDC5/irisin, and interleukin-6, which are able to regulate mental abilities [45].

With the aim of completing the MBG axis decription, it has to be noted that the gut-brain axis includes the vagus nerve (VN), a mixed nerve composed of 80% afferent and 20% efferent fibers with anti-inflammatory properties and the circumventricular organs (CO), the gut hormone signaling, the immune system, the serotonin, and the tryptophan metabolism and microbial metabolites such as short-chain fatty acids (SCFAs) [46]. The neuroactive compounds released by bacteria, such as the *γ*-aminobutyric acid (GABA), the serotonin, the dopamine, and the acetylcholine locally acting within the enteric nervous system also reaches the brain by blood [47]. Other bacterial metabolites exerting neuroactive functions include long and SCFAs [2] such as acetate, propionate, and butyrate that are important metabolites in intestinal homeostasis maintenance. The existence of a gut-brain axis has been demonstrated in Alzheimer's disease (AD). In a murine model, gut inflammation, enteric dysmotility, and intestinal AD-related protein deposition were found in early stages of the disease [48]. Similarly, Palmitoylethanolamide (PEA), a lipid mediator, has proven to counteract intestinal dysmotility associated to AD. Specifically, PEA is able to prevent glial hyperactivation and the enteric deposition of AD-related proteins, with a decreased inflammatory status [49].

## 3. Psychobiotics and Physical Exercise in Mental Disorders

With regard to psychological well-being, some gastrointestinal diseases have been recognized as triggered by biopsychosocial factors, such as the IBS, often accompanied by depression and anxiety [50], and the inflammatory bowel disease (IBD). These syndromes are influenced by an individual's stress response because of the stimulation of the sympathetic nervous system and the inhibition of the vagus [2]. Stress, anxiety, and depressed mood may be manipulated by the gut microbiome [51]. Accordingly, a double-blind randomized controlled trial (RCT) on volunteers receiving a probiotic (i.e., Probio-Stick) containing *Lactobacillus acidophilus* and *Bifidobacterium longum* during a 3-week period significantly reduced stress-induced gastrointestinal symptoms (i.e., abdominal pain and nausea/vomiting). Another RCT documented multiple benefits of *Lactobacillus plantarum* assumed 1 × 10^9^ cfu/day for 12 weeks in terms of reduced stress and anxiety [52]. The use of 24 billion cfu *Lactobacillus casei* strain Shirota (LcS) for 2 months was also shown to reduce anxiety symptoms in patients with chronic fatigue syndrome [53].

Altered gut microbial profiles have been found in some medical conditions, including psychiatric disorders [9]. Differently from healthy subjects, an increased bacterial diversity in feces of autistic children consisting of *Bacteroidetes*, *Proteobacteria*, *Actinobacteria*, and *Firmicutes* has been found [54]. A recent systematic review concluded that major depressive disorder, bipolar disorder, and schizophrenia were not characterized by differences in the number or distribution (i.e., *α*-diversity) of gut bacteria but display compositional differences compared to controls (i.e., *β*-diversity) [55]. Further, dysbiotic alterations of the gut microbiota may lead to local inflammation and increased permeability of the gastrointestinal wall leading to an augment of liposaccharides (LPS) circulation. They activate the production of systemic inflammation mediators (i.e., IL-1*β*, IL-6, IL-8 e TNF-*α*) that have been found to be higher in psychiatric patients, such as those suffering from schizophrenia [56]. High levels of IL-6 and TNF-*α* were also found in patients with bipolar disorder during both mood alterations and euthymic phases [56]. The phenomenon known as “*leaky gut*” has been proposed to shed light on major depressive disorder (MDD), too, as a proinflammatory response induced by external and internal stressors and by an increased translocation of the LPS from gram-negative bacteria [57].

Psychobiotics include a range of substances that may affect the gut-brain axis signaling, including probiotics (i.e., living microorganisms contained in food products or supplements), prebiotics (i.e., the substrate used by the host organism conferring health benefits), synbiotics (i.e., a combination of probiotics and prebiotics), and postbiotics (i.e., metabolites of bacterial fermentation and bioactive compounds) [58]. Specifically, probiotics have some effects in ameliorating certain psychopathological symptoms by improving intestinal homeostasis. Their supplementation may serve in adaptation to exercise as aiding muscle recovery and supporting skeletal muscle [59]. Akkasheh et al. [60] found a decreased Beck Depression Inventory (BDI) total score after complementary treatment with probiotic administration (i.e., *Lactobacillus acidophilu, Lactobacillus casei,* and *Bifidobacterium bifidum*, 2 × 10^9^ cfu/g) for 8 weeks in patients with MDD. Similar results on the same psychodiagnostic scale were reached by Kazemi et al. [61] by using a formula containing freeze-dried *Lactobacillus helveticus* and *Bifidobacterium longum* at a dosage of ten billion colony-forming units (i.e., ≥10 × 10^9^ CFU) per 5 g. sachet on an 8-week treatment. Further, a change in the 17-item Hamilton Depression Rating Scale score and BDI score from baseline to week 8 were found after an adjunctive therapy of *Clostridium butyricum* MIYAIRI 588 in patients with treatment-resistant MDD [62]. Finally, substantial shifts to the microbial community in response to dietary patterns may cause important health implications, as reported in attention deficit hyperactivity disorder [63].

Beyond probiotics assumption, physical exercise has been shown to be a significant factor causing changes in qualitative and quantitative composition of the gut microbiome [64]. Specifically, studies reported that exercise may have positive effects on gut microbiota increasing butyrate-producing bacteria (i.e., *Roseburia hominis*, *Faecalibacterium pausnitzii*, and *Ruminococcaceae*), for diversity and balance between beneficial and pathogenic bacterial communities, and colon health [65, 66]. Moderate intensity physical exercise (i.e., <70% VO2max) provide beneficial effects to the human body, thanks to physiological and metabolic adaptations, with changes in skeletal muscle including mitochondrial biogenesis, concentration of the substrate transporting proteins, activity of the enzymes involved in metabolic pathways, and glycogen storage in the muscle [67] whereas intensive physical exercise (i.e., >70% VO2max) may disturb the homeostasis of the gut microbiota [13] by increasing gastrointestinal wall permeability and by diminishing the gut mucus thickness, potentially favoring pathogens to enter the bloodstream, thus increasing inflammation levels [29]. A parallelism can be drawn with regard to physical activity and mood, because moderate exercise has been shown to be useful in supporting affective state while intense exercise may lead to its deterioration [68]. An adequate level of physical activity increases the synaptic transmission of monoamines, releases endorphins, and improves positive emotions experienced after the exercise [68]. A recent systematic review has shown that combined resistance and aerobic training or aerobic training alone may have positive effect on the microbiota, incrementing some bacteria phyla (i.e., *Bacteroidetes, Firmicutes, and Proteobacteria*) although further research with higher methodological rigor is needed to better understand such a relationship [9]. Studies on physical activity in clinical samples pointed out that it can normalize reduced levels of brain-derived neurotrophic factor (i.e., BDFN), with neuroprotective effects on the brain while other investigations have documented anxiolytic effects of aerobic exercise for induced-panic symptoms [69]. In addition, the aforementioned effects of physical activity on the gut microbiota suggest that the better the composition of the microbiota, the greater the capacity for nutrient degradation. Greater nutrient degradation results in both greater macronutrient availability and glycemic control [70]. All these effects have an impact on the neuronal activity. For example, it has been demonstrated that athletes present an enriched profile of SCFAs (especially, acetate, propionate, and butyrate), due to the specific activity of the microbacteria modulated by physical activity [66]. Subsequently, the produced SCFAs act as a nutritional substrate to support microglia function and this leads to an improvement in mental abilities [71].

## 4. Conclusion and Implications for Clinical Neuroscience Research and Therapeutics

The exact composition of the gut microbiota is different for each individual, and it is still unclear what may constitute a healthy profile. Determining a healthy microbiota should be a prerequisite for evaluating clinical deviations and proceeds towards tailored interventions. Such a kind of observation can be taken into consideration by clinicians to study in-depth the modification of the microbiota, also in the case of psychotropic medication orally taken [72, 73]. Alterations of the gut microbiota composition have been found in some psychiatric disorders but heterogeneity in terms of ethnicity, age, comorbidities, medication, unhealthy nutrition, antibiotics use, aging, and environmental factors, complicates a definite description [74, 75]. All these factors should be considered when planning a study on the microbiota and interpreting results. The probiotics could be useful when ingested in a definite quantity through the interaction with commensal gut bacteria and their benefits are mediated by several mechanisms referred to the hypothalamic-pituitary-adrenal (i.e., HPA) axis, the immune response and inflammation, and the production of neurohormones and neurotransmitters [76]. The rebalancing of a dysbiotic flora through the use of psychobiotics represents a therapeutic goal as a complementary intervention to standard care, especially for depressive symptoms [77, 78], even if additional RCTs in clinical populations are warranted to better evaluate their efficacy. Further, the stimulation of the vagus nerve is also recognized as an effective neurophysiological treatment in depression [79] because of the possibility to alter the cerebrospinal fluid concentration of neurotransmitter or their metabolites (e.g., GABA, and 5h1AA), and influence the functionality of certain brain regions that are dysregulated in mood disorders (i.e., orbitofrontal cortex, insula, thalamus and hypothalamus, and cingulate and hippocampus) [80]. Food hygiene and probiotics supplementations should be carefully taken into account as an integrative aspect of a multidimensional intervention on psychiatric disorders, due to the fact that many pathologies report unbalanced diet (e.g., consumption of highly saturated fats and sugar, low fiber intake, etc.) or difficulties in weight management, potentially impacting microbiota profile [81]. To this end, psychoeducational interventions focused on balanced diet adherence for a healthy lifestyle may improve quality of life of psychiatric patients, and nutritional psychiatry should be called into question with the final aim of improving clinical outcomes of standard treatments.

Evidence of positive effects of physical activity in mental disorders are limited to date. Nevertheless, outdoor activities are associated with greater feelings of revitalization, increased energy and positive engagement with tension, confusion, and anger decrease [82] and should be considered in structured psychotherapeutic protocols for depression, such as cognitive-behavioral ones implementing motor activation [83, 84]. Physical exercise further improves behavioral outcomes in psychiatric disorders by psychological mechanisms of body scheme reinforcement, changes in health attitudes, greater awareness in proprioception, and counteracts inactivity as a typical feature of patients with depression [85]. However, physical exercise as a psychosocial additional intervention for psychiatric disorders needs to be better investigated by rigorous RCTs [86] because of paucity and methodological limitations of the existing studies.

In the opinion of the authors, evidence on probiotics supplementation and physical activity in depressed mood treatment as adjunctive strategy in the context of a multidimensional intervention including pharmacology and psychotherapy is somewhat interesting. However, advances on MGB axis research have to be carefully integrated with clinical data derived from blood tests, neuropsychological and psychodiagnostic measures, and functional status examination, to better depict the relationship among the microbiota, the brain, and the musculoskeletal system.
